# Work-Related Mental Health Under COVID-19 Restrictions: A Mini Literature Review

**DOI:** 10.3389/fpubh.2021.788370

**Published:** 2021-11-24

**Authors:** Wei Liu, Yingbo Xu, Danni Ma

**Affiliations:** ^1^Business School, Qingdao University, Qingdao, China; ^2^UTS Business School, University of Technology Sydney, Ultimo, NSW, Australia

**Keywords:** COVID-19 restrictions, work-related mental health, employees, developing and developed countries, social distancing, remote working

## Abstract

Coronavirus Disease 2019 (COVID-19) restrictions, including national lockdown, social distancing, compulsory quarantine, and organizational measures of remote working, are imposed in many countries and organizations to combat the coronavirus. The various restrictions have caused different impacts on the employees' mental health worldwide. The purpose of this mini-review is to investigate the impact of COVID-19 restrictions on employees' mental health across the world. We searched articles in Web of Science and Google Scholar, selecting literature focusing on employees' mental health conditions under COVID-19 restrictions. The findings reveal that the psychological impacts of teleworking are associated with employees' various perceptions of its pros and cons. The national lockdown, quarantine, and resuming to work can cause mild to severe mental health issues, whereas the capability to practice social distancing is positively related to employees' mental health. Generally, employees in developed countries have experienced the same negative and positive impacts on mental health, whereas, in developing countries, employees have reported a more negative effect of the restrictions. One explanation is that the unevenly distributed mental health resources and assistances in developed and developing countries.

## Introduction

The COVID-19 pandemic has raised dramatic changes in the working landscape worldwide. The governments and organizations have implemented a series of emergency packages, such as mandatory lockdown, social distancing, and quarantine, to curb the coronavirus from further spreading and emergency measures on physically resuming to work after the lockdown. Besides, a vast majority of employees have also switched immediately to working from home, known as “teleworking,” “remote working,” or “smart working” (1), responding to the directives of their organizations. Working under these restrictions produces unprecedented challenges to employees, among which is to adapt to the abrupt shifts in working conditions quickly. Even though they are, to a large extent, physically protected, concerns about their mental health have sharply arisen in the extant literature (2).

The phenomenon of implementing COVID-19 restrictions is novel to the research disciplines of both organization studies and public health. Notably, as a containment strategy to prevent employees from physical harm, the implementation of COVID-19 restrictions unexpectedly brings up mental health issues on them. For example, some employees are found experiencing higher psychological distress (e.g., a negative dimension of mental health) following the work-from-home (WFH) guidelines of their organizations (3, 4). However, the complex impact of applying COVID-19 restrictions on work-related mental health is largely understudied compared to the vast majority of literature focusing on the impact of the outbreak of COVID-19 (5). Besides, in contrast to the tremendous psychological problems derived from the COVID-19 pandemic (6), the psychological impact of COVID-19 restrictions remains unclear. Extant literature presents mixed findings on the impact of COVID-19 restrictions on mental well-being (7). Even though the COVID-19 restrictions have placed strain on different cohorts (8), we suggest paying more attention to employees—a representative group experienced both national and organizational restrictions during the pandemic. The restriction-induced job insecurity and financial hardship add more challenges and are likely to escalate some psychological symptoms further (9). Consequently, our research is concerned about how the responses of governments and organizations to the COVID-19 pandemic affect employees' mental health.

The purpose of this mini-review is to examine the psychological impact of the various epidemic-related restrictions on employees. To this end, we systematically select the studies regarding employees' mental health under national-level restrictions, such as mandatory lockdown, quarantine, social distancing, resuming to work, and teleworking guidelines of companies or public organizations (e.g., public schools). The various restrictions may lead to different psychological impacts due to their inherent differences. The review identifies the variety of COVID-19 restrictions and the associated psychological impact on employees. In contrast to the primary concentration on healthcare workers or general populations, we argue that employees should be a chief concern as their mental well-being is strongly associated with economic development and labor cost to society (10).

The review contributes to understanding the impact of emergency measures (e.g., COVID-19 restrictions) on work-related mental health. Chiefly, we categorize various COVID-19 restrictions and their psychological impact on employees. In doing so, the review straightforwardly presents the consequences of the various restrictions on the work-related mental health of employees. Additionally, the review documents evidence from diverse countries to track the influence of specific restrictions on employees' mental health and statistically analyses the variations on each epidemic-related restriction's impact in developed and developing countries. The review responds to the call for attention on employees' mental well-being associated with the health emergencies at the workplace during the COVID-19 pandemic (11). More importantly, the review suggests practitioners (e.g., managers, policymakers) fully consider the complexity and consequences of applying COVID-19 restrictions. Timely mental health support is urgently needed to assist employees who have been psychologically struggling under the ongoing implementation of COVID-19 restrictions.

## Methods

For a wide-ranging and disciplined collection of the literature on employees' mental health and COVID-19 restrictions, we performed systematic literature searching for the relevant articles. The search scope comprises four factors: time span, keywords, language, and databases. Primarily, we selected articles in the English language, and published between January 2020 and August 2021. The combined terms of “COVID-19 restrictions,” “employees” or “workers,” and “mental health” or “psychological well-being” are used as the keywords searching in the databases “Web of Science” and “Google Scholar.”

**Figure 1:**
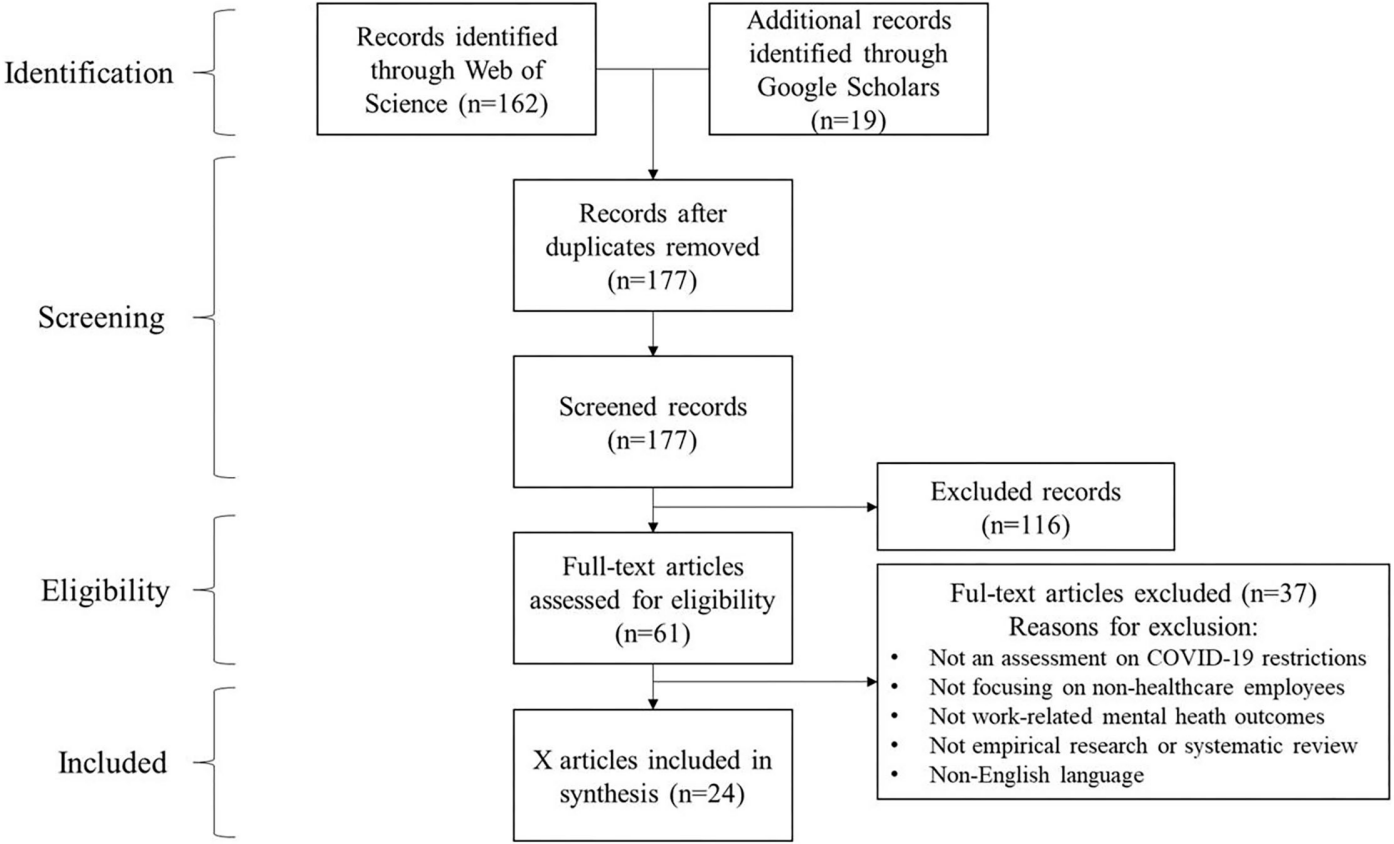
Literature search strategy, following PRISMA flow diagram (12).

The initial search resulted in the identification of 177 related articles. Article selection was conducted by two authors in an independent manner. The first round of manual screening is based on article title and abstract. We excluded the non-empirical research, narrative literature reviews, and the articles without considering COVID-19 restrictions and employees' mental health. Besides, the research that exclusively focuses on healthcare workers is also excluded. The disputes on the inclusion of each article were jointly discussed and solved with the contribution of all co-authors. The first round of screening resulted in 61 articles. Sequentially, the author proceeds to extensive reading of the introduction and conclusion of these articles. Finally, 37 articles have been excluded from the review because their focuses have no relation to the review topic. In the end, 24 highly relevant research articles have been confirmed. The literature selection strategy was visualized in Figure 1.

## Results

The findings cover four government responses, including national lockdown, resuming to work with approval, social distancing, mandatory quarantine, and a broadly used organization response in many industries—remote working. We separate remote working from other national restrictions for two reasons. Primarily, remote working as an organizational work arrangement has emerged prior to the pandemic (13). Second, remote working does not apply to the general workforce as the other government responses do. For example, grocery retailers, restaurant employees do not apply to the remote working guidelines. It is only workable for certain groups of the workforce who can manage their work flexibly with no restrictions to location.

### National-Level Restrictions

COVD-19 restrictions are a series of non-pharmaceutical measures carried out to prevent the spread of the virus. Governments worldwide have declared strict national-level measures to prevent transmission of the coronavirus. The findings reveal that among those country-level health emergencies, mandatory lockdown, quarantine, social distancing, and resuming to work are recognized as highly associated with the psychological impact of employees.

### Lockdown

Scholars describe nationwide lockdown as at the forefront of various restrictions, that is, through measures like closing non-essential businesses, limiting public transportation. Lockdown has proven effective in suspending the spread of the coronavirus but at the expense of psychological effects (14).

Both negative and positive psychological impacts of national lockdown are reported in the reviewed studies. Apouey et al. (15) have found that gig economy workers, especially food delivery bikers, are less stressed during the lockdown due to their working conditions, allowing them to keep physical activities and enjoy the beautiful urban view delivering food to customers. In contrast, drivers, also as gig economy workers, show no significant increase in anxiety and stress during the national lockdown in France. Abbas et al. (16) also report reduced stress of employees during the lockdown in Pakistan. Nevertheless, the studies carrying out in developing countries (i.e., Indian) show more negative psychological reactions than positive outcomes. The sudden changes in the routine of working and living lead to employees' psychological stress, social disconnectedness, a sense of loneliness in Indian (17), and depression in Pakistan (18).

The underlying reasons for employees' mental health issues are primarily due to the lockdown-induced fear of job insecurity (16), financial losses (17), and excessive exposure to misinformation while using social media to keep social connections (18). Therefore, scholars suggest several practical interventions, such as social support, timely and sufficient mental health assistance, to mitigate the emerged psychological symptoms during the lockdown period (16, 17).

### Resuming to Work

Due to effective control of the pandemic, a growing number of employees in many countries have been physically attending to the workplace. In China, part of the workforce has resumed work after seeking approval from the government since the ending of an extended nationwide lockdown on February 10, 2020 (19). By June 2020, many states in the U.S. also allowed restaurants to reopen and employees resuming work (20). Around the same time in Bangladesh, some financial institutions are permitted to operate with limited hours (21).

Surprisingly, the easing restrictions are more associated with adverse psychological reactions. Employees show psychological symptoms, including psychological distress (3, 21, 22), depression, anxiety, stress, worries, insomnia, somatization (19, 20, 23), and emotional reactions (24). Scholars have found that the fear of contracting the coronavirus is the chief concern of employees, especially those who cannot avoid face-to-face interactions during work (e.g., bank employees, restaurant workers, teachers) (3).

Nevertheless, workplace measures, such as workplace hygiene and indoor mask mandates (19, 22, 23), are important to influence employees' mental health conditions. Scholars report strong evidence that the deficiency of workplace measures is associated with higher stress levels of employees (23), especially for the frontline workforce, including bank employees (21), school teachers (24), and restaurant workers (3). On the contrary, sufficient and clear workplace guidelines can vastly reduce the psychological distress of the workforce (19, 22). Besides, social support is also essential to release employees' worry about unemployment (20). Bufquin et al. (3) report that furloughed or unemployed individuals has experienced a lower level of psychological distress than working employees in the restaurant industry in the U.S. because they received social support (e.g., financial compensation, tax credits) from the federal government.

Employees' mental health conditions show more negative than positive consequences under the easing of restrictions. Studies carried out in developed countries (i.e., USA, Demark, Japan) show two adverse outcomes (2/7 articles) and one positive outcome (1/7 articles); in contrast, studies in developing countries (i.e., China, Bangladesh) show three adverse outcomes (3/7 articles) and one positive outcome (1/7 articles).

### Social Distancing

Social distancing is also a national measure to avoid gathering among people during the pandemic. Scholars suggest that social distancing has generated net social benefits of $5.16 trillion to curb coronavirus transmission in the U.S. (25). Scholars also call attention to the psychological effect of implementing social distancing nationwide (26). In a recent study, Lan et al. (27) report that social distancing can ease the depression and anxiety of employees in the grocery retail industry, where the likelihood of close contact with other people is high.

### Quarantine

Governments worldwide have imposed various quarantine restrictions for different groups of individuals during the pandemic. For example, individuals with positive COVID-19 tests are isolated in hospitals (28); international travelers are compulsorily quarantined in designated hotels for 14 days after entry to a country (29). Most quarantine-related studies are concerned about the psychological impact on vulnerable groups, including healthcare workers, children and older adults (30). Teng et al. (31) investigate the employees who are working in the designated hotels for quarantine accommodation, finding that that the quarantine hotel employees have experienced mental health burden due to the augmented risk of contact guests suspected to have or infected by COVID-19 and the increased workload of operating a quarantined hotel.

### Organizational-Level Restrictions

Apart from the government mandates, teleworking, as a prominent measure initiated by many companies and organizations, also places strain upon employees' mental well-being (32).

### Remote Working

Remote working is not a new phenomenon for employees and companies (13). As a working practice for some professionals to voluntary work offsite from the office, remote working initially attempts to provide flexible work-life arrangements (7). Compared to conventional telework, pandemic-induced remote working is mandatory in nature (33), and companies and organizations have never before enforced employees to work full time at home simultaneously in a global range (32). On the one hand, using telecommunication devices to complete work has significantly minimized the risk of spreading the virus through regular close contact with others (4). On the other hand, as a growing phenomenon, employees' psychological reactions to remote working emerge as a fundamental problem (4).

Scholars report psychological symptoms induced by remote working during the pandemic ranging from stress, emotional distress, emotional exhaustion, and anxiety to depression (4, 32, 34). Employees' mental health issues are associated with their perceptions of the pros and cons of telework. The acknowledged advantages of teleworking include saved commuting time, flexible working conditions, and lower risk of COVID-19 infection, whereas the dark sides are technical issues, blurred work-life boundaries, distractions, and social disconnection (7, 35). In contrast to the general employees, some groups of employees are more easily to develop negative perceptions than positive ones, such as autistic employees (35), teachers, and university employees (7, 36), due to the challenges of adaptation to teleworking.

On top of that, the strict management control and monitor (32), deteriorated work engagement (37), and excessive job demand (34) can aggravate employees' mental health psychological symptoms during remote working. Also, employees in developed countries (i.e., Italy, Finland, Germany, U.S., Canada, Norway, U.K., and Australia) report similar positive (4/11 articles) and negative (3/11 articles) psychological impacts. In contrast, those in developing countries (i.e., Israel, Egypt, Indonesia, Chile) show more negative (3/11 articles) psychological impacts of remote working.

A more specific description of these included articles is shown in Table 1.

**Table 1:** Description of included articles.

**Level of COVID-19 restrictions**	**COVID-19 restrictions**	**Work-related mental health**	**Country**	**Factors considered**	**Main results**	**Methods**	**Population setting/*N* (if available)**	**References**
Organizational level	Remote working	Emotional distress	Israel	Perceived advantage/ disadvantage of telework	Autistic employees show a marginally significant deterioration in their mental health because they are more vulnerable to the disadvantages of remote working than the advantages	Mixed methods (survey and qualitative interview)	Autistic employees (disadvantaged population in the workforce)/*N* = 23 (quant), *N* = 10 (qual)	Goldfarb et al. (35)
		Occupational stress	Italy	Perceived advantage/ disadvantage of telework	The mobile workers show reduced stress due to saved commuting time, flexibility, and work-life balance in teleworking	Cross sectional/phone survey	Mobile workers/*N* = 51	Moretti et al. (1)
		stress	Italy	Management control	Remote working causes a sudden shift of management controls, including the increased number of digital meetings, more demanding from supervisors and clients, and constraining control, which increases the stress levels of the PSF employees	Field study/interview	PSF employees/*N* = 15	Delfino and van der Kolk (32)
		Perceived stress	Italy	Perceived advantage/ disadvantage of telework	Teachers are affected most in their mental health comparing the other three professional categories due to the less perceived benefits of teleworking	Cross sectional/online survey	Professional employees (practitioners, managers, executive employees, teachers)/*N* = 628	Mari et al. (38)
		Emotional exhaustion, psychological well-being	Egypt	Perceived advantage/ disadvantage of telework	Employees developing positive perceptions of remote working have better psychological well-being. In contrast, employees who have negative perceptions of telework show emotional exhaustion	Cross sectional/online survey	Employees/*N* = 318	Mostafa (7)
		The Depression, Anxiety, and Stress Scale	Indonesia	Reduced pandemic-related uncertainty	Employees show minimal to slight acute depression (18.4%), anxiety (46.5%), and stress (13.1%) during remote working	Cross sectional/online survey	Employees/*N* = 472	Sutarto et al. (4)
		Psychological distress	Finland	Work engagement	Remote working leads to an increase of psychological distress due to the deterioration in work engagement	Longitudinal/online survey	General employees/*N* = 965	Oksa et al. (37)
		Emotional exhaustion	Germany and USA	Excessive job demand	Excessive job demands in telework lead to employees' emotional exhaustion through the increased number of unfinished tasks	Online survey	Employees in Germany/*N* = 168	Koch and Schermuly (34)
							Employee in the USA/*N* = 292	
		Stress	Canada	Perceived advantage/ disadvantage of telework	Employees' stress level is lower due to the reduced risk of exposure to the virus in teleworking	Cross sectional/online survey	General employees/*N* = 459	Parent-Lamarche and Boulet (39)
		Distress, psychosocial well-being, quality of life, loneliness	Norway, UK, USA, and Australia	Perceived advantage/ disadvantage of telework	The remote working employees show better mental health conditions than those who were unemployed across the four countries. Employees from Norway show better mental health conditions than those in UK, USA, and Australia due to their preference for teleworking	Cross sectional/online survey	Individuals that were 18 years of age and over/*N* = 3,810	Ruffolo et al. (40)
		Psychological distress (i.e., depression, anxiety, stress)	Chile	Perceived advantage/ disadvantage of telework	A majority of university employees have experienced a high level of stress due to the challenges of adaptation to remote working	Cross sectional/online survey	University employees/*N* = 192	Gutierrez and Gallardo (36)
National level	Lockdown	Stress and anxiety	France	Perceived advantage/ disadvantage of the lockdown	Drivers show no significant increase in stress and anxiety levels, and bikers even show lower stress levels during the lockdown compared to other precarious workers. Bikers' lower stress is due to the characteristics of their working conditions, such as physical activities and the chance to enjoy the beauty of the urban view	Mixed method (interviews and longitudinal/online survey)	Gig economy workers (i.e., bikers and drivers) /(qualitative respondents/*N* = 94; quantitative participants/*N* = 137)	Apouey et al. (15)
		Psychological well-being, psychological distress	USA	Social support	Working employees have a higher level of psychological distress than furloughed or laid-off employees due to the heightened likelihood of exposure to the virus.	Cross sectional/online survey	Restaurant employees/*N* = 585	Bufquin et al. (3)
					Unemployed individuals show no significant difference in psychological well-being than employed due to the government's social support			
		6-item general health questionnaire	Pakistan	Social support	Job insecurity is adverse to employees' mental health when social support is low	Time-lagged field survey	Hospitality employees/*N* = 272	Abbas et al. (16)
		Psychological stress, social disconnectedness, and sense of loneliness	Indian	Mental health assistance	The majority of the respondents have experienced desolation and disconnectedness during the lockdown due to financial losses and blurred work-life boundaries	Qualitative interview	Middle-level employees in private sector organizations/*N* = 22	Varshney (17)
		Depression	Pakistan	Social media usage	The excessive social media usage during social distancing of the pandemic lead to employee depression due to overexposure to misinformation	Longitudinal/online survey	University employees and IT employees/*N* = 267	Majeed et al. (18)
	Returning to working physically at workplace after lockdown	Psychological distress	Bangladesh	Workplace measures, social support	A majority of bank employees are likely to experience a moderate to severe level of psychological distress due to the lack of personal protective equipment when they were returning to work after a national lockdown	Cross sectional/online survey	Private commercial bank employees/*N* = 120	Rana and Islam (21)
		Depression, anxiety, stress, and insomnia	China	Workplace measures	Employees report a low prevalence of mental health issues after returning to work due to workplace measures	Cross sectional/online survey	Employees/*N* = 1,323	Tan et al. (19)
		Anxiety, depression, insomnia, and somatization	China	social support, mental health assistance	Employees show a prevalence of anxiety (12.7%), depression (13.5%), insomnia (20.7%) and somatization (6.6%) after returning to work due to the worry about unemployment	Cross sectional/online survey	Employees/*N* = 709	Song et al. (20)
		Emotional reactions	Demark	Perceived advantage/ disadvantage of telework	Remote-working teachers show higher levels of worry than those teaching at school when they return to teaching physically at school	Cross sectional/online survey	Public school teachers/*N* = 2,665	Nabe-Nielsen et al. (24)
		Psychological distress	Japan	Workplace measures	The number of workplace measures is positively associated with employees' work-related mental health	Cross sectional/online survey	Full-time employees/*N* = 1,448	Sasaki et al. (22)
		Stress and worries	Hong Kong, China	Workplace measures	The deficiency of workplace measures has caused an increase in employees' stress levels	Cross sectional/online survey	Employees/*N* = 1,049	Ho et al. (23)
	Social distancing	Depression and anxiety	USA	Workplace measures, mental health assistance	Grocery retail employees who can practice social distancing at the workplace have experienced low anxiety and depression	Cross sectional/on site survey	Grocery retail employees/*N* = 104	Lan et al. (27)
	Quarantine	The Depression, Anxiety, and Stress Scale	China	Workplace measures, mental health assistance	During the pandemic, the temporary quarantine accommodation restrictions harmed the mental health of quarantine hotel employees in China due to the augmented risk of contact guests suspected to have or infected by COVID-19 and the raised workload of operating a quarantined hotel	Survey	Quarantine hotel employees/*N* = 170	Teng et al. (31)

## Discussion

The review aimed to address the influence of the implementation of COVID-19 restrictions on employees' mental health. The results show that COVID-19 restrictions can have both negative and positive psychological impacts on employees. The underlying reason for the increased psychological well-being of employees is primarily associated with the minimized fear of contracting the virus. In contrast, the mild to severe psychological symptoms induced by implementing these restrictions arise due to multiple reasons ranging from individual, practical, to social factors.

First, some employees are found more likely to experience deteriorated mental health than others during the restrictions. For example, autistic employees are more vulnerable to the disadvantages of remote working than its advantages (35). Similarly, some frontline workers (except healthcare workers in this review) such as quarantine hotel employees, bank employees, teachers, and university employees show psychological symptoms resuming to work due to the challenges of increased risks of contracting the virus (21, 24, 31). In contrast, gig economy workers, especially food delivery bikers, have lower stress during the lockdown due to the chance to do physical activities and enjoy the beautiful urban view while working (15). These findings suggest that the psychological reactions of vulnerable employees and frontline employees are more intense than others.

Second, this mini-review highlights several practical factors, including workplace measures and management practices, essential to mental health under COVID-19 restrictions. For example, deficiency of workplace measures undermines employees' mental health, leading to psychological symptoms such as stress and worries (23), depression, anxiety, stress, and insomnia (19). In contrast, clear and comprehensive workplace guidelines can reduce employees' psychological distress (22). Similarly, strict management control and excessive job demand during teleworking can lead to emotional exhaustion (34) and stress (32). Also, reduced work engagement during teleworking can cause employees' psychological distress (37).

Besides, in line with other studies (11), this mini-review also presents the importance of social factors in mitigating or exacerbating employees' psychological reactions under implementing COVID-19 restrictions. In this regard, social support, such as financial support programs, can prevent symptoms of psychological distress, especially for furloughed or dismissed employees during the pandemic (3). Similarly, psychological support programs, such as online mental health assistance or counseling, are helpful to alleviate the psychological issues of employees. The review suggests that sufficient social support, both financially and psychologically, plays an essential role in safeguarding employees' mental health during implementing COVID-19 restrictions.

Additionally, the studies reviewed consistently report more adverse impacts on employees' mental health than positive effects. Consistent with Flores et al. (41), our mini-review suggests that regardless of the success of cubing the spread of COVID-19, public health restrictions must be coupled with the efforts to shape proper interventions managing its psychological impacts on employees. The adverse outcomes are evident when it comes to compare and contrast with the data sourced countries. In 24 reviewed articles, studies conducted in developed countries report six negative and positive impacts, respectively, in contrast to 10 adverse outcomes of the research in developing countries. One plausible explanation is the lack of online mental health care resources in developing countries (42).

## Conclusion

Despite the recent growth of this field, attention to the psychological impacts of COVID-19 restrictions remains low in contrast to the primary concentration on the effect of the pandemic *per se*. Most studies are mainly concerned about the general population rather than employees (11), and research exhibiting employees' psychological reactions toward various COVID-19 restrictions is still limited. Based on the available 24 articles focusing on several pandemic restrictions, namely, national lockdown, resuming to work, social distancing, quarantine, and remote working, our mini-review reveals more adverse psychological impacts than positive ones on employees, especially in developing countries. We suggest that proper interventions must be arranged to safeguard employees' mental health.

## Author Contributions

WL, YX, and DM wrote the initial drafts, reviewed the manuscript, and provided comments and feedback. All authors contributed to the article and approved the submitted version.

## Conflict of Interest

The authors declare that the research was conducted in the absence of any commercial or financial relationships that could be construed as a potential conflict of interest.

## Publisher's Note

All claims expressed in this article are solely those of the authors and do not necessarily represent those of their affiliated organizations, or those of the publisher, the editors and the reviewers. Any product that may be evaluated in this article, or claim that may be made by its manufacturer, is not guaranteed or endorsed by the publisher.
